# Investigation of Parasitic Nematodes Detected in the Feces of Wild Carnivores in the Eastern Qinghai-Tibet Plateau, China

**DOI:** 10.3390/pathogens11121520

**Published:** 2022-12-12

**Authors:** Qilu Chen, Xu Wang, Chunyang Li, Weiping Wu, Kaige Zhang, Xueying Deng, Yi Xie, Yayi Guan

**Affiliations:** 1 NHC Key Laboratory of Parasite and Vector Biology, WHO Collaborating Centre for Tropical Diseases, National Center for International Research on Tropical Diseases, National Institute of Parasitic Diseases, Chinese Center for Disease Control and Prevention (Chinese Center for Tropical Diseases Research), Shanghai 200025, China; 2 Tianjin Cancer Hospital Airport Hospital, Tianjin 300308, China

**Keywords:** nematode, Qinghai-Tibet Plateau, Uncinaria stenocephala, *Toxascaris sp.*, Vulpes vulpes, Vulpes ferrilata

## Abstract

Wildlife shares grazing areas with herders in the eastern Qinghai-Tibet Plateau, and humans can be infected by zoonotic nematodes through direct contact with animals or contaminated water. In this study, fecal samples (*n* = 296) from wild carnivores were collected to explore the infection rate and molecular genetic characteristics of nematodes by stratified random sampling in the survey areas. Host species and the nematodes they carried were then identified using 16S rRNA and 18S rRNA gene sequencing, respectively. Statistical analysis, neutrality tests, genetic diversity analysis and Bayesian inferred trees were performed to complete the study. In total, 10 species of nematodes were detected in 240 feces from six species of carnivores identified (including dominant *Vulpes ferrilata* and *Vulpes vulpes*), namely *Uncinaria stenocephala*, *Toxascaris sp*., *Crenosoma vulpis*, *Parapharyngodon bainae*, *Oesophagostomum muntiacum*, *Aspiculuris tetraptera*, *Mastophorus muris*, *Nematodirus spathiger*, *Muellerius capillaris*, and *Molineus patens*. Among these nematodes, *U. stenocephala* (35.83%, 86/240) and *Toxascaris sp.* (14.58%, 35/240) were detected at higher rates than the other nematodes (χ^2^ = 516.909, *p* < 0.05). Of 17 and 18 haplotypes were found based on the ITS1 gene for *U. stenocephala* and *nad*1 gene for *Toxascaris sp.*, respectively. For the first time, using molecular methods, we report the infection of *V. ferrilata* by *U. stenocephala*, a potential zoonotic parasite, and suggest *Toxascaris sp.* may be a newly discovered nematode that lives within the fox intestine.

## 1. Introduction

Nematodes are unsegmented helminths widely distributed in freshwater, brackish water, soil, plants, and animals [1]. According to the reports, over 25,000 species of nematodes have been described so far, 12,000 of which are vertebrate parasitic nematodes [2]. For humans, some of the most prevalent but neglected tropical diseases are caused by nematode infections (e.g., ascariasis, hookworm disease, whipworm disease, lymphatic filariasis, and onchocerciasis), which lead to a decline in health and productivity, adversely affecting socio-economic development [3]. The statistics based on the global burden of diseases caused by soil-transmitted nematodes showed that ascariasis, trichuriasis, and hookworm disease, were estimated to result in 4.5 and 39 million disability-adjusted life-years (DALYs) [4]. Among them, certain nematodes are among the most common infectious pathogens in developing countries with poor sanitation, potentially affecting the psychological and physical development of children by causing anemia, malnutrition, stunting, and cognitive impairment [5,6]. According to the third national survey of important zoonotic diseases in China in 2015, Sichuan Province is a highly endemic area for soil-derived nematodes with a 23.55% infection rate, and herders are a key population experiencing soil-derived nematode infections [7].

Wild carnivores carry a variety of nematodes, some of which are zoonotic parasites that can cause human infection through contaminated water, food, or direct contact, and nematodiasis including toxocariasis and hookworm diseases could be transmitted from wild hosts to humans [8]. Clinically, human infections with *Toxocara spp.* can cause covert toxocariasis, neurotoxocariasis (e.g., eosinophilic meningoencephalitis), ocular larva migrans, and visceral larva migrans, affecting millions of children and adolescents worldwide [9,10]. From 1983 to 2019, a total of 103 documented human toxocariasis cases were reported in China, mainly in the southern provinces, and the seroprevalence of *Toxocara spp.* among children increased from 12.14% in 1993 to 19.3% in 2015 [11,12]. Hookworm, a blood-sucking nematode of the mammalian digestive system, is an important cause of neonatal mortality [13]. Iron deficiency anemia is the main clinical symptom of hookworm infection, potentially leading to proteinemia and generalized edema, in addition to cutaneous larva migrans or creeping [14,15]. There were an estimated 16.97 million hookworm infections in China in 2015 [16]. In addition, Cai [17] compiled the species of parasitic infections in ruminant livestock in Qinghai Province, and found that there were 85 species in six orders, 15 families and 24 genera belonging to the phylum Nematoda, including 71 species of digestive nematodes, such as *Toxaocara vitulorum*, *Oesophagostomum asperum*, *Nematodirus sapathiger* and *Bunostomum phlebotomum* and 13 species of pulmonary nematodes, such as *Muellerius capillaris* and one species of nematode that parasitizes the transverse muscle—*Trichinella spiralis*.

However, most of the current studies about nematodes focus on livestock and humans, and the potential for wildlife transmission of zoonotic nematodes has long been overlooked [8]. In an investigation of wildlife, Eslahi et al. reported that the pooled prevalence of toxocariasis in *Canis aureus* and *Vulpes vulpes* was 23.3% and 69.4%, respectively, and a high prevalence of 9.3% for humans in Iran [18]; Waindok et al. found that the prevalence of *Toxocara canis* in red foxes and raccoon dogs was 43.8% and 33.3%, respectively, in northern Germany [19]. In China, there was a record of *Canis lupus* infected with *T. canis* in Heilongjiang Province [20]; moreover, an investigation by Xie et al. showed that three species of ascaridoid nematodes, namely *T. canis*, *Toxocara cati* and *Toxascaris lenonia* were detected from 11 species of wild animals including *Prionailurus bengalensis*, *Felis chaus*, *Catopuma temminckii*, *Alopex lagopus*, *V. vulpes*, *Panthera tigris altaica*, *Panthera tigris amoyensis*, *Panthera tigris corbetti*, *Panthera leo spelaea*, *Lynx lynx* and *Canis lupus* in Sichuan Province, China [21]. Another study showed that black bears, red foxes, and bobcats exhibited the highest parasitic hookworm species diversity, with infection by *Ancylostoma pluridentatum*, *Ancylostoma tubaeforme*, *Uncinaria stenocephala*, and *Necator americanus* [22]. In central Europe, the prevalence of hookworm infection in red foxes is as high as 67.9% [23]. In Asia, hookworm prevalence was approximately 48% in jackals and 19% in red foxes [24]. Notably, the boundary between wildlife and humans is gradually shrinking, with an increasing number of wildlife habitats intersecting with humans and living places owing to rapid population growth and urbanization [24,25]. Therefore, there is an urgent need for the investigation of parasitic nematode infection in wild animals to reduce the potential risk of nematodiasis.

The eastern Tibetan Plateau is a global biodiversity hotspot [26], where carnivores such as *V. ferrilata*, *V. vulpes*, *Panthera uncia*, *Canis lupus*, and *Ursus arctos pruinosus* are widely distributed [27]. This plateau is also an important grazing area for Tibetan sheep and yaks [28,29]. At present, the limited investigation and surveillance of parasitic infections in the region mostly target humans and livestock, with knowledge of nematode infections in local wild carnivores remaining limited. Therefore, we conducted a preliminary exploration of local wild carnivore nematode infections to establish a basis for devising disease prevention and control measures at the interface between humans, animals, and the environment.

## 2. Materials and Methods

### 2.1. Study Area

Shiqu County is located northwest of Ganzi Tibetan Autonomous Prefecture, Sichuan Province, the eastern part of the Qinghai-Tibet Plateau, at the junction of Sichuan Province, Qinghai Province, and the Tibet Autonomous Region, with an average altitude of above 4200 m, where animal husbandry is the main vocation in the alpine meadows covering the vast majority of territory [30,31]. It belongs to an area of subtropical climate, but the climate in the territory is influenced by the complex topography of the Qinghai-Tibet Plateau, showing the characteristics of a mountainous climate and continental climate of the Qinghai-Tibet Plateau [32]. 

### 2.2. Sampling

In October 2021, five townships, namely Derongma, Niga, Sexu, Luoxu, and Zhengke, in Shiqu County, were selected for investigation based on previous survey results and information on the local landscape distribution. Sampling points were selected via stratified random sampling based on the proportion of land-use area within the study area extracted from the 2020 China Land Use Remote Sensing Monitoring Data released by the Institute of Geographic Sciences and Natural Resources Research, Chinese Academy of Sciences. Sixty-seven 15 m × 200 m plots were sampled. Each sampling point was spaced 5 km apart. All fecal samples of field carnivores were thoroughly searched at the selected sites, with the fresher samples collected and stored in 50 mL centrifuge tubes and numbered using new disposable tools to avoid cross-contamination.

### 2.3. Molecular Identification

Each fresh fecal sample collected was mixed well, and 2 g of each sample was used for DNA extraction. Deionized water (50 mL) was added to each fecal sample, followed by incubation in a water bath at 80 °C for 10 min. Thereafter, the sample was filtered through two layers of gauze, and the filtrate was added into another 50 mL centrifuge tube, then centrifuged at 3000× *g* for 30 min. The supernatant was discarded, and the precipitate was retained. DNA was extracted using the QIAamp Fast DNA Stool Mini Kit as per the manufacturer’s instructions (QIAGEN, Hilden, Germany). Parallel polymerase chain reaction (PCR) tests were performed for each sample using six pairs of primer pairs. Primer synthesis was conducted by Sangong Biotech Co. Ltd. (Shanghai, China). Host universal primers (16S) were used to identify host species based on the 16S rRNA gene, and a primary screening was performed to detect nematodes using three pairs of universal parasitic primers (Nems A/B/C) targeting the 18S rRNA gene. Considering the lack of reference sequences for the amplified target fragment, the specific primers ITS1 and *nad*1 were used to enrich sequence information for *Unicinaria sp.* and *Toxascaris sp.*, respectively (see Table 1 for details).

All PCRs were carried out in a final reaction volume of 50 μL, consisting of 25 μL of Ex TaqTM PCR premix (RR902A; Takara, Dalian, China), 17 μL of RNase-free water (9012, Takara, Dalian, China), 1 μL of bovine serum albumin (2320, Takara, Dalian, China), 1 μL of each primer (10 pmol/μL) (Sangon Biotech Co. Ltd., Shanghai, China), and 2 μL template DNA. PCR reactions were performed on a Bio-Rad DNA Engine Dyad PCR instrument (Bio-Rad) with the following amplification reaction parameters: 94 °C for 5 min, followed by 35 cycles of denaturation at 94 °C for 30 s, annealing for 30 s (annealing temperature selected according to primers), extension at 72 °C for 30 s and a final extension step at 72 °C for 10 min. 

All PCR products were sequenced, and abnormal sequencing results with multiple peaks were cloned at Sangong Biotech (Shanghai) Co., Ltd. The final sequences obtained were compared with those in the National Center for Biotechnology Information (NCBI) database (https://www.ncbi.nlm.nih.gov, accessed on 15 September 2022) to identify host and parasite species.

### 2.4. Statistics

SPSS (version 25.0; IBM Corp., Armonk, NY, USA) was used for the statistical analysis of data, with indicators such as frequency and rate used to describe host species identification and parasite detection. Comparisons between rates or composition ratios were performed using the chi-squared test at a significance level of *p* = 0.05. Comparisons between groups were performed using the Z test and Bonferroni correction. Fisher’s exact test was used to compare data that did not meet the requirements for chi-squared tests.

### 2.5. Phylogenetic Analyses

Reference sequences for the *nad*1 gene of *Uncinaria stenocephala*, ITS1 gene of *Toxascaris sp.*, and 18S rRNA gene of *Crenosoma vulpis* were searched in NCBI (see Additional file: Table S1 for details). All the sequences were first imported into MEGA 7 for alignment and trimming [37]. Thereafter, the ordered sequences were input into DNASP 6 to obtain haplotypes and perform neutrality tests, including haplotype diversity, nucleotide diversity, Tajima’s D, and Fu’s Fs [38]. The haplotype network graphs were mapped using Network 10.2.0.0 (http://www.fluxus-engineering.com) (accessed on 27 September 2022). Bayesian phylogenetic trees for *U. stenocephala*, *Toxascaris sp.*, and *Crenosoma vulpis* were constructed to analyze the relationship between the nematodes obtained in this survey and other regions. The best nucleotide substitution models were screened using jModeltest 2.1.10 [39]. Phylogenetic trees were constructed using MrBayes 3.2.4 [40]. Markov chain Monte Carlo (MCMC) data simulation was used to estimate the posterior probabilities with 10,000 generations of operations, the sampling frequency was set to 10, and the first 25% of aging samples were discarded. Finally, Bayesian trees were compiled and processed using FigTree 1.4.3 (http://tree.bio.ed.ac.uk/software/figtree, accessed on 27 September 2022). 

## 3. Results

### 3.1. Host Species Composition

**Figure 1:**
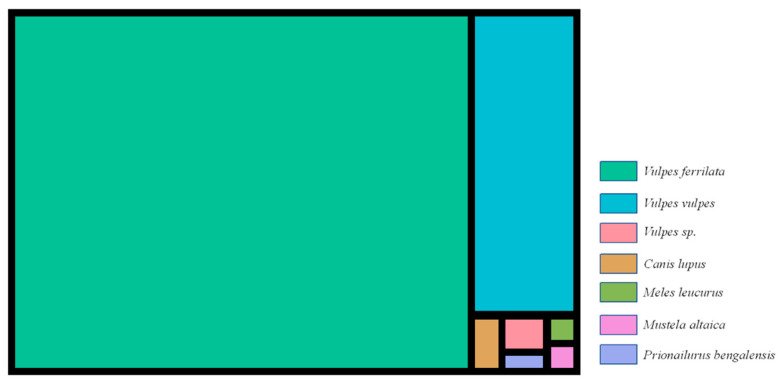
Results of 16S rRNA sequencing of fecal samples for host identification. Note: *Vulpes sp.* indicates that 16S rRNA gene fragments of both *Vulpes ferrilata* and *Vulpes vulpes* were detected in one sample.

Of the 296 fecal samples initially recognized by the investigators as wild canids, 240 were successfully identified after DNA extraction (81%). Six carnivorous species belonging to the families of Canidae, Mustelidae, and Felidae were identified, namely *Vulpes ferrilata* (81.25%, 195/240), *Vulpes vulpes* (15.83%, 38/240), *Vulpes sp.* (0.83%, 2/240), *Canis lupus* (0.83%, 2/240), *Meles leucurus* (0.42%, 1/240), *Mustela altaica* (0.42%, 1/240), and *Prionailurus bengalensis* (0.42%, 1/240) (Figure 1). The foxes, especially *V. ferrilata*, were dominant among carnivores in the area (Figure 1).

### 3.2. Nematode Detections

A total of 10 nematode species were detected in 240 host samples using three parasitic universal primers (NemA/B/C): *Uncinaria stenocephala*, *Toxascaris sp.*, *Crenosoma vulpis*, *Parapharyngodon bainae*, *Oesophagostomum muntiacum*, *Aspiculuris tetraptera*, *Mastophorus muris*, *Nematodirus spathiger*, *Muellerius capillaris*, and *Molineus patens* (accession numbers of 18S rRNA gene in NCBI database: OP788051–OP788057, OP788064), with an overall detection rate of 57.92% (139/240, 95% CI: 51.67–64.16%). All nematodes were detected in fox feces (*V. ferrilata* and *V. vulpes*). *Uncinaria stenocephala* (35.83%, 95% CI: 29.77–42.25%) and *Toxascaris sp.* (14.58%, 95% CI: 10.12–19.05%) were detected at a higher rate than other nematodes in all carnivore feces (χ^2^ = 516.909, *p* < 0.05), and the detection rate of nematodes in *V. ferrilata* feces was significantly higher than that in *V. vulpes* feces (χ^2^ = 4.200, *p* < 0.05), especially in the case of *U. stenocephala* (χ^2^ = 4.903, *p* < 0.05). In addition, the detection rate of *Molineus patens* between *V. ferrilata* and *V. vulpes* was significantly different (χ^2^ = 0.000, *p* < 0.05, Fisher’s exact test). Further details are provided in Table 2.

### 3.3. Nucleotide Polymorphisms and Haplotype Network Graph Construction Results

**Figure 2:**
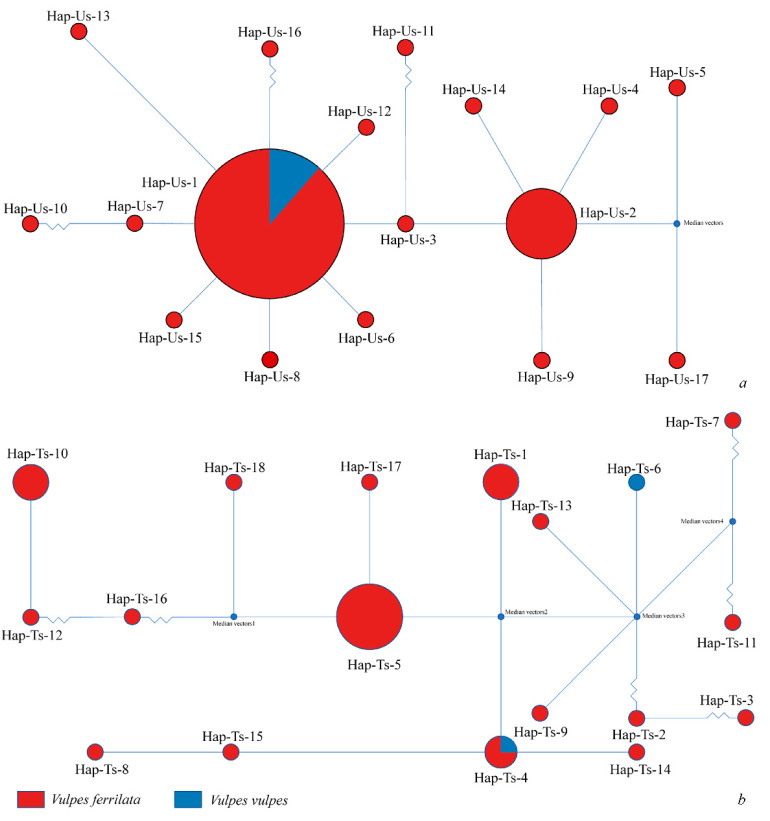
The haplotype network of *Uncinaria stenocephala* based on 122 ITS1 gene sequences and *Toxascaris sp.* based on 45 *nad*1 gene sequences. (**a**) Colors represent different hosts, the size of the circle is proportional to the number of sequences with that haplotype, the distances between circles are proportional to the base differences between haplotypes, and median vectors were inferred using NETWORK software. Hap-Us-1 differs from Hap-Us-16 by six bases, Hap-Us-3 differs from Hap-Us-11 by two bases, and Hap-Us-7 differs from Hap-Us-10 by two bases. (**b**) Median vectors3 differs from Hap-Ts-2 by three bases, Hap-Ts-2 differs from Hap-Ts-3 by three bases, Median vectors4 differs from Hap-Ts-11 by two bases, Median vectors4 differs from Hap-Ts-7 by two bases, Median vectors1 differs from Hap-Ts-16 by seven bases, and Hap-Ts-16 differs from Hap-Ts-12 by eight bases.

The haplotype network graphs showed that 122 DNA sequences obtained from *U. stenocephala* were clustered as 17 haplotypes (Hap-Us-1–Hap-Us-17) based on the ITS1 gene (accession in NCBI database: OP788006–OP788023). Hap-Us-1 (number of sequences included was 87) and Hap-Us-2 (*n* = 19) were the main gene haplotypes for *U. stenocephala* in the study areas. All haplotypes were detected in the feces of *V. ferrilata*, whereas only Hap-Us-1 was isolated from *V. vulpes* (*n* = 10) (Figure 2a). Correspondingly, 18 haplotypes (Hap-Ts-1–Hap-Ts-18) were counted from 45 *nad*1 gene sequences of *Toxascaris sp.* (accession in NCBI database: OP800273–OP800283, OP805342–OP805348), with Hap-Ts-5 as the core, dominant haplotype detected in *V. ferrilata*. Hap-Ts-4 (*n* = 1) was a common haplotype shared between *Toxascaris sp.* from *V*. *ferrilata* and *V. vulpes*, and Hap-Ts-6 was found only in *V. vulpes* (Figure 2b). 

### 3.4. Nucleotide Polymorphisms

Neutral tests showed significant negative results (Tajima’s D = −2.20369, *p* < 0.05; Fu’s Fs = −12.411, *p* < 0.05) for *U. stenocephala*, suggesting that the population had a large number of low-frequency mutant genes in its gene pool and underwent an expansion after a recent bottleneck effect under significant selection pressure. Moreover, *U. stenocephala* had lower genetic diversity (Hd = 0.470 and Pi = 0.00249) than *Toxascaris sp.* (Hd = 0.836 and Pi = 0.01836). In contrast, although the neutrality tests showed negative values, there was no significant difference for *Toxascaris sp.* (Tajima’s D = −4.9277, *p* > 0.10; Fu’s Fs = −2.610, *p* > 0.10), indicating that *Toxascaris sp.* population dynamics were closer to neutrality with random evolution (see Table 3 for details).

### 3.5. Phylogenetic Tree Results

**Figure 3:**
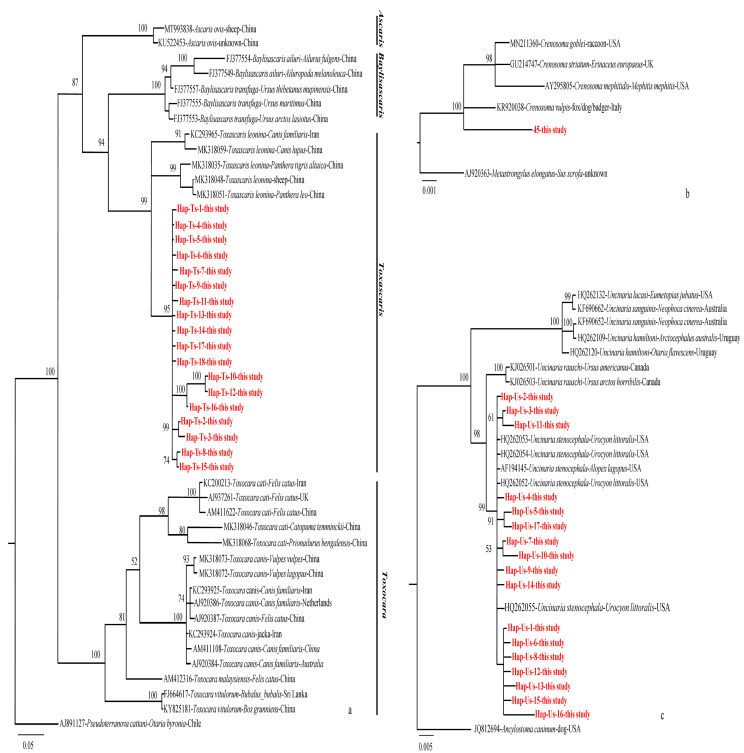
Bayesian phylogenetic trees for three nematodes detected in fox feces from Shiqu County and constructed using MrBayes 3.2.4. (**a**) Phylogenetic tree of 18 *Toxascaris sp.* haplotypes of the *nad*1 gene, with the nucleotide substitution model “HKY + I + G”. (**b**) Phylogenetic tree based on the *Crenosoma vulpis* 18S rRNA sequence, with the nucleotide substitution model “HKY + I”. (**c**) Phylogenetic tree of 17 *Uncinaria stenocephala* haplotypes of the ITS1 gene with the nucleotide substitution model “K80”. Bootstrap values are shown, and the sequences retrieved from the NCBI are shown with accession numbers.

The Bayesian tree showed that *U. stenocephala* isolates from the United States and the haplotypes in this survey were located on the same branch, with 99% support. However, some haplotypes were clustered into new sub-branches, such as Hap-Us-1, Hap-Us-6, Hap-Us-8, Hap-Us-12, Hap-Us-13, Hap-Us-15, and Hap-Us-16, which may have been formed by potential geographical divergence (Figure 3c). As shown in Figure 3a, some parasitic gene sequences obtained from this study were clustered on a sister branch of *Toxascaris leonina* and were, therefore, considered a new local species of the *Toxascaris* genus. Figure 3b shows that the genes of *Crenosoma vulpis* from this study and from a study in Italy clustered together.

### 3.6. Primers Variability Comparison

**Figure 4:**
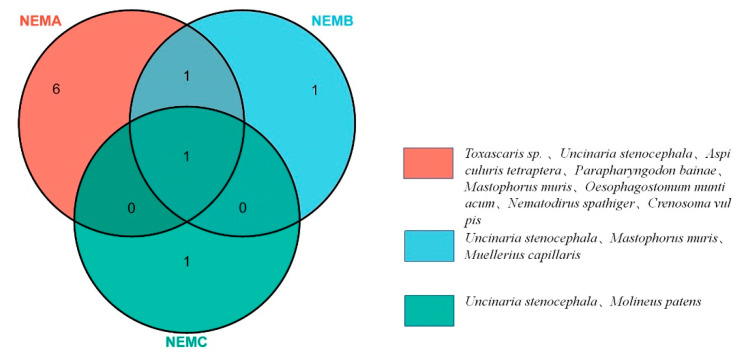
Overlap of nematode species detected using the three nematode universal primers (Nem A/B/C).

Of the 10 nematode species detected, only one (*U. stenocephala*) was detected using all three primers, and Nem A detected the largest number of nematode species (Figure 4). Statistically significant differences were observed in the detection rates of parasites using different primers. The detection rate of *Toxascaris sp.* was higher for Nem A than for Nem B and C (χ^2^ = 73.577, *p* < 0.05). The detection rates of *U. stenocephala* were higher for Nem B and Nem C than for Nem A (χ^2^ = 16.620, *p* < 0.05), and the detection rate of *Mastophorus* muris was higher for Nem A than for Nem C (χ^2^ = 9.520, *p* < 0.05, Fisher’s exact test).

## 4. Discussion

This study provides molecular evidence for the presence of local nematode-infected carnivores in the Qinghai-Tibetan Plateau. In this study, six species of carnivores were identified through the analysis of fecal DNA collected throughout Shiqu County, Ganzi Tibetan Autonomous Prefecture, the eastern Qinghai-Tibetan Plateau. Foxes, especially *V. ferrilata*, were the dominant species within the region based on fecal analysis. In the present work, the zoonotic parasite *U. stenocephala* was reported to infect *V. ferrilata* for the first time through molecular detection. The findings suggest that *Toxascaris sp.* may be a new parasitic species affecting foxes, distinct from *T. leonina* and other *Toxascaris sp.* reported worldwide.

*Uncinaria stenocephala* belongs to the family Ancylostomatidae [41], which is widely parasitic in dogs, wolves, foxes, and badgers, as well as other canids, and is distributed in regions with colder climates throughout Asia, Europe, North America, South America, Australia, and New Zealand [42]. It is among the most common hookworms infecting dogs [43]. Cats can also be infected by this parasite, but their resistance is higher than that of canines [44,45]. *U. stenocephala* eggs are transmitted via the feces of definitive canine hosts, Larvae hatch in the superficial soil layer within one day and then develop into third-stage larvae after approximately one week under ideal conditions [46,47]. Humans are infected through skin contact with infective third-stage larvae or through the consumption of raw food containing infectious third-stage larvae [48]. However, due to the lack of relevant investigations in China, only very limited data can be referred to. The earliest records were reported in Sichuan Province: *U. stenocephala* was observed in the intestines of *V. ferrilata* and *Arctonyx collaris* by morphological methods in Luhuo County and Hongyuan County in 1963 and 1981, respectively [49]. Since then, there were no reports in the literature until 2019, when *U. stenocephala* infection was determined morphologically in five of 10 dogs examined in Nangqian County, Qinghai Province [50]. In the present work, the overall detection rate of *U. stenocephala* in foxes was 36.9% (86/233), lower than rates reported from Denmark (60–86%) [51], Spain (58.2%) [52], Switzerland (64–78%) [53,54], and Italy (39–75%) [55], but comparable to that in Ireland (38%) [56]. Those data suggest that foxes are an important definitive host for *U. stenocephala and* play a key role in the process of transmission that cannot be ignored. This should be considered a warning because this parasite can cause disease in humans. Fourteen *U. stenocephala* isolates were detected from the feces of 128 patients in Iran [57]; further, 22% of prisons in Madrid, Spain, had reported *U. stenocephala* infections [58] and compared to a prevalence of 7.9% from 38 residents tested in a suburb of Buenos Aires, Argentina. While no *U. stenocephala* infection in humans has been reported in China thus far, given the high detection rate of foxes in the present investigation, it is rather likely for transmission to the local residents to have occurred. Furthermore, when patients have hookworm-related cutaneous larva migrans, the location of the larvae is usually more distant than the visible area of the lesion and cannot be diagnosed via biopsy or based only on clinical features and epidemiological history [59]. Thus, we recommend relevant diagnostic training for local physicians to raise vigilance for *U. stenocephala* infection.

It is worth mentioning that a species of *Toxascaris sp.*, suspected to be a new species of the genus *Toxascaris*, was identified in this study, with a detection rate of 14.58%. The phylogenetic tree revealed that it was closely related to *Toxascaris leonina*, currently the only known species in this genus [60]. The eggs of *T. leonina* can adapt to cold climatic conditions, survive for 40 days at −15 °C, and continue to develop to the infectious stage when the temperature returns to 25 °C, after which it infects animals via the oral route [61]. In general, canines and cats are the definitive hosts of *T. leonina*; small rodents can be their intermediate hosts, while rabbits and chickens can act as reservoir hosts [62,63]. In particular, *T. leonina* migrates into the intestinal wall only during the larval stage and has a long incubation period (48–72 days). A young host can complete growth and development before its health is affected, and thus, *T. leonina* is considered to have low pathogenicity [64]. Current research suggests that *T. leonina* is a potentially zoonotic parasite that may be responsible for eosinophilia among people in the St. Lawrence Island region of Alaska [60,63]. Whether the *Toxascaris sp.* found in this study has the same pathogenic potential as *T. leonina* requires further study.

*Crenosoma vulpis*, a parasite species with foxes as the common definitive host, also infects stray and domestic dogs as well as various other carnivores such as *Canis lupus*, *Canis aureus*, *Nyctereutes procyonoides*, *Urocyon cinereoargenteus*, *Lutra lutra* and *Meles meles*. Further, it is an important causative pathogen of chronic respiratory disease in canids [65]. Infected animals suffer from chronic respiratory diseases, exhibit poor growth, and may even die due to bronchopneumonia in severe cases [66]. Its larvae reside in gastropods, with third-stage larvae (L3) infecting the definitive host. Adult nematodes reside within the bronchi and bronchioles where they produce first-stage larvae (L1) that can be coughed up within three weeks of infection or passed from the feces of definitive hosts through swallowing [67]. The high infection rate of *Crenosoma vulpis* in *V. vulpes* has been reported in Europe and America, including 78.4% in Canada [68], 8.7–15.8% in Italy [69], and 24.6% in Hungary [70]. We found only one infection case in *V. ferrilata* in this study, which may be related to the fact that the samples collected are feces rather than lung tissues.

In addition to the above three parasites with foxes as definitive hosts, some parasites detected in this study may not be common intestinal species of foxes. *Parapharyngodon bainae* is an intestinal parasite affecting carnivorous reptiles [71]. *Oesophagostomum muntiacum* usually lives in the intestinal tract of muntjacs and can also be parasitic in primates, such as macaques [72,73]. *Aspiculuris tetraptera*, a common parasite in the digestive system of mice, is usually non-pathogenic or slightly pathogenic in mice with a normal immune system [74]. *Mastophorus muris* is a common parasitic nematode in the stomachs of rodents, and its intermediate hosts are beetles, locusts, earwigs, cockroaches and other insects [75]. *Muellerius capillaris* is a common pulmonary parasitic nematode in small ruminants, such as goats and sheep, causing respiratory distress and coughing in small ruminants, making their lungs more susceptible to bacterial infection [76,77]. *Molineus patens* is a common intestinal parasite of mustelids; it can impair the health of skunks, affecting their population size [78,79]. This observation may be related to the feeding habits of carnivores. *Vulpes ferrilata* identified in this survey mainly prey on pika and voles [80], its prey also includes *Pantholops hodgsoni*, *Lepus oiostolus*, *Phrynocephalus spp.*, *Marmota himalayana*, *Moschus spp.*, *Pseudois nayaur*, and young domestic animals [81,82]. While foxes might not be the most suitable host, the current study indicates the local presence of these parasitic nematodes.

All results of this study were obtained by molecular methods and were not supported by morphological analysis. Because, *V. ferrilata* is protected in China [83], only non-invasive methods, such as fecal sample testing, can be applied in survey studies. Further in-depth study of parasitology and host-parasite relationships should be carried out in the future. However, even with the use of molecular detection, it should be noted that the experimental results of three pairs of nematode primers showed differences in detection rates. Therefore, we suggest that parallel experiments using multiple primer pairs would be a prudent approach for future studies.

The livestock industry is the major source of income within the Ganzi Prefecture on the eastern Tibetan Plateau, and its development has led to considerable overlap between the living areas of local herders and wild habitats [84,85]. Shared grazing areas and water sources are key factors in facilitating the transmission of nematodes between wildlife and humans [86,87]. The current surveillance of foxes and humans on the Tibetan Plateau for zoonotic nematode infection focuses mainly on echinococcosis [88,89], with the transmission of some zoonotic nematodes, such as *U. stenocephala*, potentially being overlooked. Monitoring and investigation of human-animal nematodes in the eastern Tibetan Plateau need to be improved to determine the disease burden of zoonotic nematodes in the area. This would require the combined efforts of ecologists, wildlife specialists, veterinarians, human physicians, and relevant disease control personnel from a “One Health” perspective (encompassing humans, domestic animals, wildlife, and the changing ecosystems in which they live [90]) in order to better prevent the spread of zoonotic nematodes.

## 5. Conclusions

Foxes are dominant among wildlife populations on the eastern Qinghai-Tibet Plateau, especially *Vulpes ferrilata*, which exhibits a very high nematode infection rate. *Uncinaria stenocephala* was the most common zoonotic nematode, detected for the first time using molecular methods, in *V. ferrilata* at a rate of 35.83%. The *Toxcascaris sp.* found in this survey may represent a new species of the genus *Toxascaris*. Taken together, we suggest strengthening parasite monitoring among foxes on the eastern Tibetan Plateau in the future to prevent the local transmission of zoonotic nematodes. 

## Figures and Tables

**Table 1:** Information on polymerase chain reaction (PCR) primers used for molecular identification.

Primers	Classification	Names	Target Genes	Primer Sequences (From 5′ to 3′)	Amplicon Length (bp)	Annealing Temperature (°C)	Reference
Parasitic universal primers	Nematode	Nem A	18S rRNA	F/CACCCGTGAGGATTGACAG R/CGATCACGGAGGATTTTCAA	320–335	53	[33]
	Nematode	Nem B	18S rRNA	F/CGTCATTGCTGCGGTTAAAA R/CCGTCCTTCGAACCTCTGAC	380–410	55	[33]
	Nematode	Nem C	18S rRNA	F/AGTGGAGCATGCGGCTTAAT R/TGCAATTCCCTRTCCCAGTC	380–440	55	[33]
Parasitic specific primers	*Uncinaria*	ITS1	ITS1	F/TTGAACCGGGTAAAAGTCG R/CGTTTTTCATCGATACGCG	432	54	[34]
	*Toxascaris*	ND1	*nad*1	F/TTCTTATGAGATTGCTTTT R/TATCATAACGAAAACGAGG	370	50	[35]
Host universal primers	Vertebrate	16S	16S rRNA	F/GAGAAGACCCTATGGAGC R/ATAGAAACCGACCTGGAT	380	55	[36]

**Table 2:** Detection rate of nematodes in 240 fecal samples from the study area in Shiqu County.

Nematodes	Detection Rate % (No. Positive of Samples/No. Total Fecal Samples, 95% CI) Carnivores									
	*Vulpes ferrilata*	*Vulpes vulpes*	χ^2^	*p*	*Canis* *lupus*	*Vulpes * *sp.*	*Meles* *leucurus*	*Mustela * *altaica*	*Prionailurus* *bengalensis*	Total
*Toxascaris sp.*	16.92 (33/195, 11.66–22.19)	5.26 (2/38, 0.64–17.75)	3.387	0.06	0.00 (0/2)	0.00 (0/2)	0.00 (0/1)	0.00 (0/1)	0.00 (0/1)	14.58 (35/240, 10.12–19.05) ^a^
*Aspiculuris* *tetraptera*	0.51 (1/195, 0.00–1.52)	0.00 (0/38)	0.000	1.00	0.00 (0/2)	0.00 (0/2)	0.00 (0/1)	0.00 (0/1)	0.00 (0/1)	0.42 (1/240, 0.00–1.23) ^b^
*Mastophorus * *muris*	2.56 (5/195, 0.35–4.78)	7.89 (3/38, 1.66–21.38)	2.726	0.24	0.00 (0/2)	0.00 (0/2)	0.00 (0/1)	0.00 (0/1)	0.00 (0/1)	3.33 (8/240, 1.06–5.60) ^b^
*Uncinaria * *stenocephala*	40.00 (78/195, 33.12–46.88)	21.05 (8/38, 8.09–34.01)	4.903	<0.05	0.00 (0/2)	0.00 (0/2)	0.00 (0/1)	0.00 (0/1)	0.00 (0/1)	35.83 (86/240, 29.77–42.25) ^c^
*Crenosoma* *vulpis*	0.51 (1/195, 0.00–1.52)	0.00 (0/38)	0.000	1.00	0.00 (0/2)	0.00 (0/2)	0.00 (0/1)	0.00 (0/1)	0.00 (0/1)	0.42 (1/240, 0.00–1.23) ^b^
*Oesophagostomum * *muntiacum*	0.51 (1/195, 0.00–1.52)	2.63 (1/38, 0.07–13.81)	0.000	0.30	0.00 (0/2)	0.00 (0/2)	0.00 (0/1)	0.00 (0/1)	0.00 (0/1)	0.83 (2/240, 0.00–1.98) ^b^
*Nematodirus* *spathiger*	0.51 (1/195, 0.00–1.52)	0.00 (0/38)	0.000	0.05	0.00 (0/2)	0.00 (0/2)	0.00 (0/1)	0.00 (0/1)	0.00 (0/1)	0.42 (1/240, 0.00–1.23) ^b^
*Muellerius * *capillaris*	0.00 (0/195)	2.63 (1/38, 0.07–13.81)	0.000	0.16	0.00 (0/2)	0.00 (0/2)	0.00 (0/1)	0.00 (0/1)	0.00 (0/1)	0.42 (1/240, 0.00–1.23) ^b^
*Molineus * *patens*	0.00 (0/195)	5.26 (2/38, 0.64–17.75)	0.000	<0.05	0.00 (0/2)	0.00 (0/2)	0.00 (0/1)	0.00 (0/1)	0.00 (0/1)	0.83 (2/240, 0.00–1.98) ^b^
*Parapharyngodon* *bainae*	1.03 (2/195, 0.00–2.44)	0.00 (0/38)	0.000	1.00	0.00 (0/2)	0.00 (0/2)	0.00 (0/1)	0.00 (0/1)	0.00 (0/1)	0.83 (2/240, 0.00–1.98) ^b^
Total	62.56 (122/195, 55.77–69.36)	44.74 (17/38, 28.62–61.70)	4.200	<0.05	0.00 (0/2)	0.00 (0/2)	0.00 (0/1)	0.00 (0/1)	0.00 (0/1)	57.92 (139/240, 51.67–64.16)

^a, b, c^ represent results of the post-hoc tests; *p* values were adjusted by Bonferroni correction and compared with 0.05 level; values with the same superscript letter indicate no significant difference between the two groups.

**Table 3:** Diversity and neutral indices of the *Uncinaria stenocephala* ITS1 gene and the *Toxascaris sp. nad*1 gene in Shiqu.

Nematodes	No. of Sequences	No. of Haplotypes	Haplotype Diversity	Nucleotide Diversity	Tajima’s D	Fu’s Fs
*Uncinaria stenocephala*	122	17	0.470 ± 0.052	0.00249 ± 0.00037	−2.20369, *p* < 0.01	−12.411, *p* < 0.01
*Toxascaris sp.*	45	18	0.836 ± 0.048	0.01836 ± 0.00305	−0.49277, *p* > 0.10	−2.610, *p* > 0.10

## Data Availability

Not applicable.

## Supplementary Materials

The following supporting information can be downloaded at: https://www.mdpi.com/article/10.3390/pathogens11121520/s1, Table S1 Accession numbers of reference sequences for the *nad*1 gene of *Uncinaria stenocephala*, ITS1 gene of *Toxascaris sp.*, and 18S rRNA gene of *Crenosoma vulpis.*

## References

[1] Poinar G.J. (2012). Nematoda (Roundworms). eLS..

[2] Morand S., Bouamer S., Hugot J.-P., Serge M., Salah B., Jean-Pierre H. (2005). Nematodes. Micro Mammals and Macroparasites: From Evolutionary Ecology to Management.

[3] Barry M.A., Simon G.G., Mistry N., Hotez P.J. (2013). Global trends in neglected tropical disease control and elimination: Impact on child health. Arch. Dis. Child..

[4] Knopp S., Steinmann P., Keiser J., Utzinger J. (2012). Nematode infections: Soil-transmitted helminths and trichinella. Infect. Dis. Clin. N. Am..

[5] Sorobetea D., Svensson-Frej M., Grencis R. (2018). Immunity to gastrointestinal nematode infections. Mucosal. Immunol..

[6] Lustigman S., Prichard R.K., Gazzinelli A., Grant W.N., Boatin B.A., McCarthy J.S., Basáñez M.G. (2012). A research agenda for helminth diseases of humans: The problem of helminthiases. PLoS Negl. Trop. Dis..

[7] Chen Y.D., Zhou C.H., Zhu H.H., Huang J.L., Duan L., Zhu T.J., Qian M.B., Li S.Z., Chen H.G., Cai L. (2020). National survey on the current status of important human parasitic diseases in China in 2015. Chin. J. Parasitol. Parasit. Dis..

[8] Otranto D., Deplazes P. (2019). Zoonotic nematodes of wild carnivores. Int. J. Parasitol. Parasites Wildl..

[9] Rostami A., Ma G., Wang T., Koehler A.V., Hofmann A., Chang B.C.H., Macpherson C.N., Gasser R.B. (2019). Human toxocariasis—A look at a neglected disease through an epidemiological ‘prism’. Infect. Genet. Evol..

[10] Hotez P.J., Wilkins P.P. (2009). Toxocariasis: America’s most common neglected infection of poverty and a helminthiasis of global importance?. PLoS Negl. Trop. Dis..

[11] Wang S., Li H., Yao Z., Li P., Wang D., Zhang H., Xie Q., Zhang Z., Li X. (2020). *Toxocara* infection: Seroprevalence and associated risk factors among primary school children in central China. Parasite.

[12] Kong L., Peng H.J. (2020). Current epidemic situation of human toxocariasis in China. Adv. Parasitol..

[13] Brooker S., Hotez P.J., Bundy D.A. (2008). Hookworm-related anaemia among pregnant women: A systematic review. PLoS Negl. Trop. Dis..

[14] Wang C.H., Lee S.C., Huang S.S., Chang L.C. (2011). Hookworm infection in a healthy adult that manifested as severe eosinphilia and diarrhea. J. Microbiol. Immunol. Infect..

[15] Zibaei M., Nosrati M.R.C., Shadnoosh F., Houshmand E., Karami M.F., Rafsanjani M.K., Majidiani H., Ghaffarifar F., Cortes H.C.E., Dalvand S. (2020). Insights into hookworm prevalence in Asia: A systematic review and meta-analysis. Trans. R. Soc. Trop. Med. Hyg..

[16] Chen Y.D., Qian M.B., Zhu H.H., Zhou C.H., Zhu T.J., Huang J.L., Li Z.J., Li S.Z., Zhou X.N., Group on National Survey of Important Human Parasitic Diseases in China (2021). Soil-transmitted helminthiasis in China: A national survey in 2014–2015. PLoS Negl. Trop. Dis..

[17] Cai J.Z. (2006). Classification of Nematode in Ruminantfrom Qinghai Province. Chin. J. Parasitol. Parasit. Dis..

[18] Eslahi A.V., Badri M., Khorshidi A., Majidiani H., Hooshmand E., Hosseini H., Taghipour A., Foroutan M., Pestehchian N., Firoozeh F. (2020). Prevalence of *Toxocara* and *Toxascaris* infection among human and animals in Iran with meta-analysis approach. BMC Infect. Dis..

[19] Waindok P., Raue K., Grilo M.L., Siebert U., Strube C. (2021). Predators in northern Germany are reservoirs for parasites of One Health concern. Parasitol. Res..

[20] Chang Q.C., Gao J.F., Sheng Z.H., Lou Y., Zheng X., Wang C.R. (2015). Sequence variability in three mitochondrial genes among four roundworm species from wild animals in China. Mitochondrial DNA.

[21] Xie Y., Chen Z., Zhao B., Niu L., Gu X., Yang G. Characterization of Roundworms from Eleven Species of Captive Wild Canin and Feline Animals, in Sichuan Provinces, China, Using Mitochondrial and Nuclear Genes. Proceedings of the 11th China Symposium on Young Workers of Parasitology.

[22] Seguel M., Gottdenker N. (2017). The diversity and impact of hookworm infections in wildlife. Int. J. Parasitol. Parasites Wildl..

[23] Karamon J., Dabrowska J., Kochanowski M., Samorek-Pierog M., Sroka J., Rozycki M., Bilska-Zajac E., Zdybel J., Cencek T. (2018). Prevalence of intestinal helminths of red foxes (*Vulpes vulpes*) in central Europe (Poland): A significant zoonotic threat. Parasites Vectors.

[24] Kimpston C.N., Hatke A.L., Castelli B., Otto N., Tiffin H.S., Machtinger E.T., Brown J.D., Van Why K.R., Marconi R.T. (2022). High Prevalence of Antibodies against Canine Parvovirus and Canine Distemper Virus among Coyotes and Foxes from Pennsylvania: Implications for the Intersection of Companion Animals and Wildlife. Microbiol. Spectr..

[25] Treves A., Karanth K.U. (2003). Human-carnivore conflict and perspectives on carnivore management worldwide. Conserv. Biol..

[26] Myers N., Mittermeier R.A., Mittermeier C.G., Fonseca G., Kent J.M.J.N. (2000). Biodiversity hotspots for conservation priorities. Nature.

[27] Dai Y., Xue Y., Hacker C.E., Zhang Y., Zhang Y., Liu F., Li D. (2020). Human-carnivore conflicts and mitigation options in Qinghai province, Chian. J. Nat. Conser..

[28] Li W., Cao W., Wang J., Li X., Xu C., Shi S.J.E.E. (2017). Effects of grazing regime on vegetation structure, productivity, soil quality, carbon and nitrogen storage of alpine meadow on the Qinghai-Tibetan Plateau. Ecol. Eng..

[29] Miao F., Guo Z., Xue R., Wang X., Shen Y. (2015). Effects of grazing and precipitation on herbage biomass, herbage nutritive value, and yak performance in an alpine meadow on the Qinghai-Tibetan Plateau. PLoS ONE.

[30] Hao L., Yuan D., Guo L., Hou W., Mo X., Yin J., Yang A., Li R. (2020). Molecular detection of Bartonella in ixodid ticks collected from yaks and plateau pikas (*Ochotona curzoniae*) in Shiqu County, China. BMC Vet. Res..

[31] Wang D., Wu J., Wang Y., Ji Y. (2020). Finding High-Quality Groundwater Resources to Reduce the Hydatidosis Incidence in the Shiqu County of Sichuan Province, China: Analysis, Assessment, and Management. Expos. Health.

[32] Shao Xinning S.D., Huang Q., Li S., Yao M. (2019). Fast surveys and molecular diet analysis of carnivores based on fecal DNA and metabarcoding. Biodiv. Sci..

[33] Cannon M.V., Bogale H., Rutt L., Humphrys M., Korpe P., Duggal P., Ravel J., Serre D. (2018). A high-throughput sequencing assay to comprehensively detect and characterize unicellular eukaryotes and helminths from biological and environmental samples. Microbiome.

[34] Catalano S., Lejeune M., van Paridon B., Pagan C.A., Wasmuth J.D., Tizzani P., Duignan P.J., Nadler S.A. (2015). Morphological variability and molecular identification of *Uncinaria spp.* (Nematoda: Ancylostomatidae) from grizzly and black bears: New species or phenotypic plasticity?. J. Parasitol..

[35] Mikaeili F., Mirhendi H., Mohebali M., Hosseini M., Sharbatkhori M., Zarei Z., Kia E.B. (2015). Sequence variation in mitochondrial *cox*1 and *nad*1 genes of ascaridoid nematodes in cats and dogs from Iran. J. Helminthol..

[36] Xiong M., Shao X., Long Y., Bu H., Zhang D., Wang D., Li S., Wang R., Yao M. (2016). Molecular analysis of vertebrates and plants in scats of leopard cats (*Prionailurus bengalensis*) in southwest China. J. Mammal..

[37] Kumar S., Stecher G., Tamura K. (2016). MEGA7: Molecular Evolutionary Genetics Analysis Version 7.0 for Bigger Datasets. Mol. Biol. Evol..

[38] Rozas J., Ferrer-Mata A., Sánchez-DelBarrio J.C., Guirao-Rico S., Librado P., Ramos-Onsins S.E., Sánchez-Gracia A. (2017). DnaSP 6: DNA Sequence Polymorphism Analysis of Large Data Sets. Mol. Biol. Evol..

[39] Darriba D., Taboada G.L., Doallo R., Posada D. (2012). jModelTest 2: More models, new heuristics and parallel computing. Nat. Methods.

[40] Ronquist F., Huelsenbeck J.P. (2003). MrBayes 3: Bayesian phylogenetic inference under mixed models. Bioinformatics.

[41] Nadler S.A., Adams B.J., Lyons E.T., DeLong R.L., Melin S.R. (2000). Molecular and morphometric evidence for separate species of *Uncinaria* (Nematoda: Ancylostomatidae) in California sea lions and northern fur seals: Hypothesis testing supplants verification. J. Parasitol..

[42] Bowman D.D., Montgomery S.P., Zajac A.M., Eberhard M.L., Kazacos K.R. (2010). Hookworms of dogs and cats as agents of cutaneous larva migrans. Trends Parasitol..

[43] Postigo I., Martínez J., Cardona G., Fernández-Pérez I., Guisantes J.A. (2003). *Uncinaria stenocephala*: Antigenic characterization of larvae and adults worms using sera from naturally infected dogs. Exp. Parasitol..

[44] Rodan I., Sparkes A.H., Susan E.L. (2012). The Cat.

[45] Datz C., Peterson M.E., Kutzler M.A. (2011). Parasitic and Protozoal Diseases. Small Animal Pediatrics.

[46] Feldmeier H., Schuster A. (2012). Mini review: Hookworm-related cutaneous larva migrans. Eur. J. Clin. Microbiol. Infect. Dis..

[47] Heukelbach J., Feldmeier H. (2008). Epidemiological and clinical characteristics of hookworm-related cutaneous larva migrans. Lancet Infec. Dis..

[48] Matthews M., Vanlier C., Montjoye L., Baeck M. (2020). A creeping holiday souvenir: About a misleading case of hookworm folliculitis *J*. Trave. Med..

[49] Wu J., Chen D.M., Ma F.H. (1982). Studies on the Tibetan fox and pig badger *Uncinario stenocephala*. Chin. J. Wild..

[50] Zhang Z.P., Hu G.W., Zhao Q.B., Li J., Shen Y.L., Zhou L., Li S., Cai J.S. (2019). *Uncinaria stenocephala* found in the Sanjiangyuan area. China Anim. Husb. Vet. Med..

[51] Al-Sabi M.N., Halasa T., Kapel C.M. (2014). Infections with cardiopulmonary and intestinal helminths and sarcoptic mange in red foxes from two different localities in Denmark. Acta Parasitol..

[52] Criado-Fornelio A., Gutierrez-Garcia L., Rodriguez-Caabeiro F., Reus-Garcia E., Roldan-Soriano M.A., Diaz-Sanchez M.A. (2000). A parasitological survey of wild red foxes (*Vulpes vulpes*) from the province of Guadalajara, Spain. Vet. Parasitol..

[53] Hofer S., Gloor S., MÜLler U., Mathis A., Hegglin D., Deplazes P. (2000). High prevalence of *Echinococcus multilocularis* in urban red foxes (*Vulpes vulpes*) and voles (*Arvicola terrestris*) in the city of Zürich, Switzerland. Parasitology.

[54] Reperant L.A., Hegglin D., Fischer C., Kohler L., Weber J.-M., Deplazes P. (2007). Influence of urbanization on the epidemiology of intestinal helminths of the red fox (*Vulpes vulpes*) in Geneva, Switzerland. Parasitol. Res..

[55] Di Cerbo A.R., Manfredi M.T., Bregoli M., Cova M. (2008). Wild carnivores as source of zoonotic helminths in north-eastern Italy. Helminthologia.

[56] Stuart P., Golden O., Zintl A., de Waal T., Mulcahy G., McCarthy E., Lawton C. (2013). A coprological survey of parasites of wild carnivores in Ireland. Parasitol. Res..

[57] Ghadirian E. (2007). Human Infection with *Uncinaria* in North of Iran. Iranian J. Parasitol..

[58] Alonso-Sanz M., Chaves F., Dronda F., Catalán S., González-López A. (1995). Intestinal parasitoses in the prison population in the Madrid area (1991–1993). Enferm. Infecc. Microbiol. Clin..

[59] Baple K., Clayton J. (2015). Hookworm-related cutaneous larva migrans acquired in the UK. BMJ Case. Rep..

[60] Jin Y.C., Li X.Y., Liu J.H., Zhu X.Q., Liu G.H. (2019). Comparative analysis of mitochondrial DNA datasets indicates that *Toxascaris leonina* represents a species complex. Parasites Vectors.

[61] Rostami A., Riahi S.M., Fallah Omrani V., Wang T., Hofmann A., Mirzapour A., Foroutan M., Fakhri Y., Macpherson C.N.L., Gasser R.B. (2020). Global Prevalence Estimates of *Toxascaris leonina* Infection in Dogs and Cats. Pathogens.

[62] Laatamna A., Baroudi D., Samari H., Ziane H., Alim O., Telibi M., Taoussi D. (2021). First report on occurrence of zoonotic helminth *Toxocara canis*, *Toxascaris leonina* and *Ancylostoma caninum* in domestic dogs from province of Djelfa, Algeria. Ann. Parasitol..

[63] Rausch R.L., Fay F.H. (2011). Toxascaris leonina in Rodents, and Relationship to Eosinophilia in a Human Population. Comp. Parasitol..

[64] Parsons J.C. (1987). Ascarid Infections of Cats and Dogs. Vet. Clin. N. Am. Small. Anim. Pract..

[65] Conboy G. (2004). Natural infections of *Crenosoma vulpis* and *Angiostrongylus vasorum* in dogs in Atlantic Canada and their treatment with milbemycin oxime. Vet. Rec..

[66] Hanson K.B. (1933). Tests of the efficacy of single treatments with trachéal brushes in the mechanical removal of lungworms from foxes. J. Am. Vet. Med. Assoc..

[67] Colella V., Mutafchiev Y., Cavalera M.A., Giannelli A., Lia R.P., Dantas-Torres F., Otranto D. (2016). Development of *Crenosoma vulpis* in the common garden snail *Cornu aspersum*: Implications for epidemiological studies. Parasites Vectors.

[68] Nevárez A., López A., Conboy G., Ireland W., Sims D. (2005). Distribution of *Crenosoma vulpis* and *Eucoleus aerophilus* in the lung of free-ranging red foxes (*Vulpes vulpes*). J. Vet. Diagn. Investig..

[69] Latrofa M.S., Lia R.P., Giannelli A., Colella V., Santoro M., D’Alessio N., Campbell B.E., Parisi A., Dantas-Torres F., Mutafchiev Y. (2015). *Crenosoma vulpis* in wild and domestic carnivores from Italy: A morphological and molecular study. Parasitol. Res..

[70] Tolnai Z., Szell Z., Sreter T. (2015). Environmental determinants of the spatial distribution of *Angiostrongylus vasorum*, *Crenosoma vulpis* and *Eucoleus aerophilus* in Hungary. Vet. Parasitol..

[71] Pereira F.B., Sousa B.M., Lima Sde S. (2011). A new species of Pharyngodonidae (Nematoda) of *Tropidurus torquatus* (Squamata:Tropiduridae) from Brazil. J. Parasitol..

[72] Setsuda A., Kato E., Sakaguchi S., Tamemasa S., Ozawa S., Sato H. (2019). *Chabaudstrongylus ninhae* (Trichostrongylidae: Cooperiinae) and *Oesophagostomum muntiacum* (Chabertiidae: Oesophagostominae) in feral alien Reeves’s muntjacs on Izu-Oshima Island, Tokyo, Japan. J. Helminthol..

[73] Medkour H., Amona I., Laidoudi Y., Davoust B., Bitam I., Levasseur A., Akiana J., Diatta G., Pacheco L., Gorsane S. (2020). Parasitic Infections in African Humans and Non-Human Primates. Pathogens.

[74] Mustakdir Z., Akbari R.A., Retnani E.B., Tiuria R. (2022). *Aspiculuris tetraptera* Infection in Mice: Parasite degree and Differential Leukocyte. J. Ris. Vet. Indones..

[75] Neupane B., Miller A.L., Evans A.L., Olsson G.E., Hoglund J. (2018). Seasonal variation of *Mastophorus muris* (Nematoda: Spirurida) in the water vole Arvicola amphibius from southern Sweden. J. Helminthol..

[76] Pybus M.J., Shave H. (1984). *Muellerius capillaris* (Mueller, 1889) (Nematoda: Protostrongylidae): An unusual finding in Rocky Mountain bighorn sheep (*Ovis canadensis canadensis Shaw*) in South Dakota. J. Wild. Dis..

[77] Foreyt W.J., Jenkins E.J., Appleyard G.D. (2009). Transmission of lungworms (*Muellerius capillaris*) from domestic goats to bighorn sheep on common pasture. J. Wild. Dis..

[78] Zalewski A., Kołodziej-Sobocińska M., Bartoń K.A. (2022). A tale of two nematodes: Climate mediates mustelid infection by nematodes across the geographical range. Int. J. Parasitol. Parasites Wildl..

[79] Vergles Rataj A., Posedi J., Zele D., Vengust G. (2013). Intestinal parasites of the red fox (*Vulpes vulpes*) in Slovenia. Acta Vet. Hung..

[80] Wang Z., Wang X., Lu Q. (2007). Selection of land cover by the Tibetan fox *Vulpes ferrilata* on the eastern Tibetan Plateau, western Sichuan Province, China. Acta Theriol..

[81] Shengwu Z. (1985). Feeding information of Tibetan fox. Acta Terminol. Sin..

[82] Liu Q., Harris R.B., Wang X. (2010). Food habits of the Tibetan fox (*Vulpes ferrilata*) in the Kunlun Mountains, Qinghai Province, China. Mamm. Biol..

[83] Wang Z.-H., Wang X.-M., Chmura A. (2008). Den Habitat Characteristics of Tibetan Foxes (*Vulpes ferrilata*) in Shiqu County, Sichuan Province, China. Zool. Stud..

[84] Deng Z.J. (2012). Analysis on Anim alHusbandry Status and the Feasibility of Developm ent Characteristic Breedingin GanziState. Mod. Agric. Sci. Technol.

[85] Craig P.S., Giraudoux P., Wang Z.H., Wang Q. (2019). Echinococcosis transmission on the Tibetan Plateau. Adv. Parasitol..

[86] Walker J.G., Morgan E.R. (2014). Generalists at the interface: Nematode transmission between wild and domestic ungulates. Int. J. Parasitol. Parasites Wildl..

[87] Wells K., Gibson D.I., Clark N.J., Ribas A., Morand S., McCallum H.I. (2018). Global spread of helminth parasites at the human-domestic animal-wildlife interface. Glob. Chang. Biol..

[88] Fu M., Han S., Xue C., Wang X., Liu B., Wang Y., Wang L., Wei S., Cui X., Zhang T. (2020). Contribution to the echinococcosis control programme in China by NIPD-CTDR. Adv. Parasitol..

[89] Lei Z.L., Wang L.Y. (2012). Control situation and primary task of key parasitic diseases in China. Chin. J. Parasit. Dis..

[90] Thompson R.C. (2013). Parasite zoonoses and wildlife: One Health, spillover and human activity. Int. J. Parasitol..

