# Supplementary material for: Investigation of Parasitic Nematodes Detected in the Feces of Wild Carnivores in the Eastern Qinghai-Tibet Plateau, China
Source: Pathogens. 2022 Dec 12;11(12):1520. doi: 10.3390/pathogens11121520 (PMC9785254; doi:10.3390/pathogens11121520)
Supplement: Supplementary file 1 [file pathogens-11-01520-s001.zip › pathogens-2059680-supplementary.pdf]

Table S1. Accession numbers of reference sequences for the *nad*1 gene of *Uncinaria. stenocephala*, ITS1 gene of *Toxascaris sp.*, and 18s rRNA gene of *Crenosoma vulpis*.

Toxascaris Sp. reference				*Uncinaria stenocephala* reference				*Crenosoma vulpis* reference			
Genbank accession no.	Species	Host	Country	Genbank accession no.	Species	Host	Country	Genbank accession no.	Species	Host	Country
KC200213	*Toxocara_cati*	*Felis_catus*	Iran	HQ262109	*Uncinaria_hamiltoni*	*Arctocephalus_australis*	Uruguay	MN211360	*Crenosoma goblei*	raccoon	USA
AM411622	*Toxocara_cati*	*Felis_catus*	China	HQ262120	*Uncinaria_hamiltoni*	*Otaria_flavescens*	Uruguay	GU214747	*Crenosoma striatum*	*Erinaceus europaeus*	UK
AJ937261	*Toxocara_cati*	*Felis_catus*	UK	KF690662	*Uncinaria_sanguinis_*	*Neophoca_cinerea*	Australia	AY295805	*Crenosoma meohitidis*	*Mephitis mephitis*	USA
MK318046	*Toxocara_cati*	*Catopuma_temminckii*	China	KF690652	*Uncinaria_sanguinis_*	*Neophoca_cinerea*	Australia	KR920038	*Crenosoma vulpis*	fox/dog/badger	Italy
MK318068	*Toxocara_cati*	*Prionailurus_bengalensis*	China	HQ262132	*Uncinaria_lucasi*	*Eumetopias_jubatus*	USA	AJ920363	*Metastrongylus elongatus*	*Sus scrofa*	unknown
AJ920387	*Toxocara_canis*	*Felis_catus*	China	KJ026501	*Uncinaria_rauschi*	*Ursus_americanus*	Canad				
MK318073	*Toxocara_canis*	*Vulpes_vulpes*	China	KJ026503	*Uncinaria_rauschi*	*Ursus_arctos_horribili*	Canad				
MK318072	*Toxocara_canis*	*Vulpes_lagopus*	China	HQ262053	*Uncinaria_stenocephala*	*Urocyon_littoralis*	USA				
KC293925	*Toxocara_canis*	*Canis_familiaris*	Iran	HQ262054	*Uncinaria_stenocephala*	*Urocyon_littoralis*	USA				
KC293924	*Toxocara_canis*	jackal	Iran	AF194145	*Uncinaria_stenocephala*	*Alopex_lagopus*	USA				
AM411108	*Toxocara_canis*	*Canis_familiaris*	China	HQ262052	*Uncinaria_stenocephala*	*Urocyon_littoralis*	USA				
AJ920384	*Toxocara_canis*	*Canis_familiaris*	Australia	HQ262055	*Uncinaria_stenocephala*	*Urocyon_littoralis*	USA				
AJ920386	*Toxocara_canis*	*Canis_familiaris*	Netherlands	JQ812694	*Ancylostoma_caninum*	dog	USA				
AM412316	*Toxocara_malaysiensis*	*Felis_catus*	China								

FJ664617	*Toxocara_vitulorum*	*Bubalus_bubalis*	Sri_Lanka
KY825181	*Toxocara_vitulorum*	*Bos_grunniens*	China
FJ377554	*Baylisascaris__ailuri*	*Ailurus_fulgens*	China
FJ377549	*Baylisascaris__ailuri*	*Ailuropoda_melanoleuca_*	China
FJ377557	*Baylisascaris_transfuga*	*Ursus_thibetanus_mupinensis*	China
FJ377555	*Baylisascaris_transfuga*	*Ursus_maritimus*	China
FJ377553	*Baylisascaris_transfuga*	*Ursus_arctos_lasiotus*	China
MT993838	*Ascaris_ovis*	sheep	China
HQ704901	*Ascaris_suum*	*Sus_scrofa_*	China
MK318035	*Toxascaris_leonina_*	*Panthera_tigris*	China
MK318048	*Toxascaris_leonina_*	*Lynx_lynx*	China
MK318051	*Toxascaris_leonina*	*Panthera_leo*	China
KC293965	*Toxascaris_leonina*	*Canis_familiaris_*	Iran
MK318059	*Toxascaris_leonina*	*Canis_lupus*	China
